# Interactive Effects of Dietary Lipid and Phenotypic Feed Efficiency on the Expression of Nuclear and Mitochondrial Genes Involved in the Mitochondrial Electron Transport Chain in Rainbow Trout

**DOI:** 10.3390/ijms16047682

**Published:** 2015-04-07

**Authors:** Jonathan C. Eya, Vitalis O. Ukwuaba, Rodrigue Yossa, Ann L. Gannam

**Affiliations:** 1 Department of Biology/Gus R. Douglass Institute, West Virginia State University, Institute, WV 25112, USA; E-Mails: vitaukwuaba2003@yahoo.com (V.O.U.); ryossa@wvstateu.edu (R.Y.); 2 USFWS, Abernathy Fish Technology Center, Longview, WA 98632, USA; E-Mail: ann_gannam@fws.gov

**Keywords:** dietary lipid, fish family, gene expression, mitochondria, rainbow trout

## Abstract

A 2 × 3 factorial study was conducted to evaluate the effects of dietary lipid level on the expression of mitochondrial and nuclear genes involved in electron transport chain in all-female rainbow trout *Oncorhynchus mykiss*. Three practical diets with a fixed crude protein content of 40%, formulated to contain 10% (40/10), 20% (40/20) and 30% (40/30) dietary lipid, were fed to apparent satiety to triplicate groups of either low-feed efficient (F120; 217.66 ± 2.24 g initial average mass) or high-feed efficient (F136; 205.47 ± 1.27 g) full-sib families of fish, twice per day, for 90 days. At the end of the experiment, the results showed that there is an interactive effect of the dietary lipid levels and the phenotypic feed efficiency (growth rate and feed efficiency) on the expression of the mitochondrial genes *nd1* (NADH dehydrogenase subunit 1), *cytb* (Cytochrome b), *cox1* (Cytochrome c oxidase subunits 1), *cox2* (Cytochrome c oxidase subunits 2) and *atp6* (ATP synthase subunit 6) and nuclear genes *ucp2α* (uncoupling proteins 2 alpha), *ucp2β* (uncoupling proteins 2 beta), *pparα* (peroxisome proliferator-activated receptor alpha), *pparβ* (peroxisome proliferatoractivated receptor beta) and *ppargc1α* (proliferator-activated receptor gamma coactivator 1 alpha) in fish liver, intestine and muscle, except on *ppargc1α* in the muscle which was affected by the diet and the family separately. Also, the results revealed that the expression of mitochondrial genes is associated with that of nuclear genes involved in electron transport chain in fish liver, intestine and muscle. Furthermore, this work showed that the expression of mitochondrial genes parallels with the expression of genes encoding uncoupling proteins (UCP) in the liver and the intestine of rainbow trout. This study for the first time presents the molecular basis of the effects of dietary lipid level on mitochondrial and nuclear genes involved in mitochondrial electron transport chain in fish.

## 1. Introduction

Fish oil (FO) is the main source of lipid used in fish nutrition, especially for carnivorous species. Many favorable attributes contribute to this extensive use of FO in aquaculture; FO is the best source of digestible energy, is readily accepted (palatable) by the farmed fish, contains essential fatty acids, does not affect flesh composition and taste of cultured-fish, and, as a lipid, can spare protein utilization to varying degrees [1,2,3]. The decrease in global fish oil availability, from 1.50 million tons in 1994 to 1.07 million tons in 2009 [4], due to the depletion of world fisheries resources, environmental concerns related to FO production, increase in global aquaculture production and the development of FO-based tablets for human consumption during the last two decades concur with the rise in FO cost [2,4,5]. Thus, during the same period of time, extensive scientific efforts have been deployed in searching alternative lipid sources which are readily available at lower price, in order to partially or totally substitute FO in aquafeed. Nevertheless, FO replacement in intensive aquaculture is still at its early-development stage, with limited practical results especially in the farming of carnivorous species [2,4,5].

Based on bioenergetics and zootechnical parameters such as maximum growth, survival, health characteristics and feed utilization, the minimum lipid and essential fatty acid requirements have been estimated in many fish species, in order to avoid FO wastage and to reduce aquaculture production costs [5]. In addition, several genetic selection programs have been developed in order to select and produce families of poultry and livestock species [6] as well as fish species [7,8,9,10,11] that show improved feed efficiency (FE), with high feed efficient (HFE) fish having higher body weight gain than low feed efficient (LFE) fish, at the same feed intake. In broiler nutrition, such improved phenotypic FE has contributed to the optimization of the nutrients (ingredients) used in diet formulation, and has been related to an improvement in mitochondrial function [6,12,13,14]. In fish, genetic variation has been reported in feed consumption, growth and feed efficiency, and improved growth and FE were associated to mitochondrial function in channel catfish *Ictalurus punctatus* [15,16] and rainbow trout *Oncorhynchus mykiss* [17].

Mitochondria are organelles that are found in eukaryotic cells, where they generate cellular energy in the form of adenosine triphosphate (ATP) via oxidative phosphorylation of nutrients (glucose, fatty acids and amino acids). Through this process, mitochondria produce approximately 90% of the energy in animal cells [18]. Mitochondrial electron transport chain (ETC), which is located in the inner membrane, comprises five multi-subunit enzyme complexes, complex I (NADH: ubiquinone oxidoreductase), complex II (succinate: ubiquinone oxidoreductase), complex III (ubiquinol: ferricytochrome c oxidoreductase), complex IV (cytochrome c oxidase) and ATP synthase sometimes referred to as complex V [19], as well as two mobile electron carriers, ubiquinone and cytochrome c [6,12,18,20]. These complexes play a crucial role in the mitochondrial ETC. Electrons from NADH-linked energy substrates such as malate and pyruvate and FADH2-linked energy substrates such as succinate enter the ETC via complex I and complex II, respectively; the complexes I and II then transfer the electrons to the carrier ubiquinone which in turn carriers them to complex III; complex III is responsible for electron transfer from ubiquinone to the second carrier (cytochrome c), and complex III also links this electron transfer to proton pumping from the mitochondrial matrix into the inner membrane, creating a proton-motive force that leads to ATP synthesis when the protons are translocated back into the mitochondrial matrix through complex V [6,12,18,20]. These complexes are made of subunits that are encoded by both nuclear and mitochondrial genes [20]. Therefore, the efficiency of the complexes I–V is conditioned by the expression of these nuclear and mitochondrial genes.

Using commercial diets containing different crude protein and crude lipid levels, it has been found that diet composition affects mitochondrial complex activity and the genes encoding these complexes in fish [16,17]. However, only one study has evaluated the effects of feeding graded level of FO, with a fixed crude protein level at 42%, on mitochondrial gene expression [21]. The results of this last study showed that the genes encoding complex I (*nd1*), complex III (*cytb*), complex IV (*cox1*), complex IV (*cox2*) and complex V (*atp6*) were up-regulated in the muscle and down-regulated in both the liver and intestine. The causes of such down- and up-regulation of the mitochondrial genes were not apparent. Such complex observations were also found in the broiler, and it has been suggested that nuclear transcriptional factors such as peroxisome proliferator activated receptor alpha and beta (*pparα* and *pparβ*) and PPAR-γ coactivator-1-alpha (*ppargc1α*), and uncoupling proteins (UCP) such as *ucp2α* and *ucp2β* are also involved in phenotypic expression of FE [6]. In addition, it has been well-established that dietary fatty acids influence the regulation of genes involved lipid and energy metabolism in mammals [22] and chicken [23], whereas limited information on this subject exist in fish species [24]. Presently, little is known about the extent or nature of mitochondrial function in relation to dietary lipid levels, or if dietary modifications such as an increase in dietary lipid levels associated with a decrease in dietary protein levels influences the development of superior mitochondrial functions by altering nuclear and mitochondrial protein and gene expression levels in rainbow trout. Thus, the objective of the present study was to investigate the effect of graded dietary lipid levels on the expression of mitochondrial and nuclear genes involved in mitochondrial electron transport chain in low-FE and high-FE families of rainbow trout fed a diet contained 40% dietary protein.

## 2. Results

Gene expression levels from all other treatments were expressed as fold-change difference when compared with the calibrating treatment group (F120 family fed diet 40/10).

### 2.1. Gene Expression in the Liver

#### 2.1.1. Mitochondrial Genes in the Liver

The effect of the interaction diet × family was highly significant (*p* < 0.01) on the expression of mitochondrial *nd1*, *cytb*, *cox1*, *cox2* and *atp6* genes in fish liver (Table 1). The *nd1* gene was up-regulated in the liver of all the treatment groups. The F136 fish fed diet 40/10 showed a higher *nd1* expression than F136 fish fed diet 40/30, whereas the lowest *nd1* expression was observed in F120 fish fed diet 40/10; however, there was no significant difference in *nd1* expression between the F136 fish fed diet 40/10 and the group comprising the F120 fish fed diets 40/20 and 40/30, and also between the F136 fish fed diets 40/20 and 40/30. The *cytb* gene was down-regulated in the liver of F136 fish fed diet 40/30, whereas it was up-regulated in all the other treatment groups. The F120 fish fed diet 40/20 showed the highest *cytb* expression, followed successively by F136 fish fed diet 40/10, F120 fish fed diet 40/30, F136 fish fed diet 40/20, and F120 fish fed diet 40/10, while the lowest *cytb* expression was observed in F136 fish fed diet 40/30. The *cox1* gene was down-regulated in the liver of F136 fish fed diets 40/10 and 40/20, whereas it was up-regulated in all the other treatment groups. The F120 fish fed diet 40/20 showed the highest *cox1* expression, followed successively by F120 fish fed diet 40/30, F136 fish fed diet 40/30, and F136 fish fed diet 40/20, while the lowest *cox1* expression was observed in F136 fish fed diet 40/10; however, there was no significant difference in *cox1* expression between F136 fish fed diet 40/30 and F120 fish fed diet 40/10, and between F136 fish fed diet 40/20 and F120 fish fed diet 40/10. The *cox2* gene was down-regulated in the liver of F136 fish fed diets 40/10 and 40/30, whereas it was up-regulated in all the other treatment groups. The F120 fish fed diet 40/20 showed the highest *cox2* expression, followed successively by F120 fish fed diet 40/30, F136 fish fed diet 40/20, and F120 fish fed diet 40/10, while the lowest *cox2* expression was observed in the group comprising F136 fish fed diets 40/10 and 40/30, which showed similar values. The *atp6* gene was down-regulated in the liver of F136 fish fed diet 40/10, whereas it was up-regulated in all the other treatment groups. The F120 fish fed diet 40/30 showed the highest *atp6* expression, followed successively by F136 fish fed diet 40/20, F136 fish fed diet 40/30, F120 fish fed diet 40/10, while the lowest *atp6* expression was observed in the group comprising F136 fish fed diets 40/20 and F136 fish fed diet 40/10, which showed similar values.

**Table 1:** Significant interactions between family and diet on mitochondrial gene expression in the liver of two juvenile rainbow trout *Oncorhynchus mykiss* families fed practical diets containing graded dietary lipid levels for 90 days ^1^.

Families ^2^	Practical Diets ^3^	Mitochondrial Gene Expression ^4^				
		Complex I: *nd1*	Complex III: *cytb*	Complex IV: *cox1*	Complex IV: *cox2*	Complex V: *atp6*
**Individual treatment means**						
F120	40/10	0.000 ^d^	0.000 ^e^	0.000 ^cd^	0.000 ^d^	0.000 ^d^
F120	40/20	0.455 ^ab^	0.777 ^a^	0.636 ^a^	0.937 ^a^	0.569 ^e^
F120	40/30	0.435 ^ab^	0.418 ^c^	0.392 ^b^	0.601 ^b^	0.459 ^a^
F136	40/10	0.564 ^a^	0.598 ^b^	−0.292 ^e^	−0.244 ^e^	−0.557 ^e^
F136	40/20	0.400 ^bc^	0.326 ^d^	−0.090 ^d^	0.209 ^c^	0.339 ^b^
F136	40/30	0.287 ^c^	−0.177 ^f^	0.027 ^c^	−0.198 ^e^	0.173 ^c^
**Pooled SEM**		0.133	0.025	0.031	0.022	0.036
**Main effect means**						
F120		0.296	0.398	0.340	0.513	0.343
F136		0.417	0.249	−0.118	−0.077	−0.015
	40/10	0.282	0.299	−0.146	−0.122	−0.278
	40/20	0.427	0.552	0.270	0.572	0.554
	40/30	0.361	0.120	0.210	0.202	0.316
**ANOVA: *p* values**						
**Family (F)**		0.0043	<0.0001	<0.0001	<0.0001	<0.0001
***F*-value/df ^5^**		13.31/1	51.77/1	331.62/1	1108.43/1	145.51/1
**Diet (D)**		0.0159	<0.0001	<0.0001	<0.0001	<0.0001
***F*-value/df ^5^**		5.96/2	146.35/2	106.16/2	512.26/2	229.51/2
**Interaction (FxD)**		<0.0001	<0.0001	<0.0001	<0.0001	<0.0016
***F*-value/df ^5^**		42.25/2	329.41/2	28.09/2	96.83/2	11.56/2

^1^ Means represent average values of three tanks. The LSD procedure was applied on individual means because the two-factor interaction was significant. Individual treatment means within a column followed by different superscript letters were significantly different (*p* < 0.05); ^2^ Families of fish selected experimentally for low feed efficiency (F120) and high feed efficiency (F136) by USDA-ARS National Center of Cool and Coldwater Aquaculture (NCCCWA) in Leetown, WV, USA; ^3^ Diets formulated to contain 40% protein and 10% fat (40/10), 40% protein and 20% fat (40/20) or 40% protein and 30% fat (40/30); ^4^ Gene expression (*nd1*, *cytb*, *cox1*, *cox2* and *atp6*) values are presented as fold-change (log RQ) compared to the calibrator, which is the low feed efficiency family fed diet with lowest dietary fat level (family 120 fed diet 40/10); and ^5^ *F*-values/degree of freedom (df) for family, diet and interaction, respectively.

#### 2.1.2. Nuclear Genes in the Liver

The effect of the interaction diet × family was highly significant (*p* < 0.01) on the expression of nuclear *ucp2α*, *ucp2β*, *pparα* and *pparβ* genes, and was significant (*p* < 0.05) on *ppargc1α* gene in fish liver (Table 2). The *ucp2α* gene was up-regulated in the liver of all the treatment groups. The F120 fish fed diet 40/20 showed the highest *ucp2α* expression, followed successively by the group comprising F120 fish fed diet 40/30 and F136 fish fed diet 40/20, the group of F136 fish fed diets 40/10 and 40/30, whereas the lowest *ucp2α* expression was observed in F120 fish fed diet 40/10. The *ucp2β* gene was up-regulated in the liver of all the treatment groups. The F120 fish fed diet 40/20 showed the highest *ucp2β* expression, followed successively by F136 fish fed diet 40/10, F136 fish fed diet 40/20, the group comprising F136 fish fed diet 40/30 and F120 fish fed diet 40/30, while the lowest *ucp2β* expression was observed in F120 fish fed diet 40/10. The *pparα* gene was up-regulated in the liver of all the treatment groups. The F120 fish fed diet 40/30 showed the highest *pparα* expression, followed successively by F120 fish fed diet 40/20, F136 fish fed diet 40/10, and the group comprising F136 fish fed diets 40/20 and 40/30, while the lowest *pparα* expression was observed in F120 fish fed diet 40/10. The *pparβ* gene was down-regulated in the liver of F136 fish fed diet 40/10, whereas it was up-regulated in all the other treatment groups. The group of fish comprising F120 fish fed diets 40/20 and 40/30 showed the highest *pparβ* expression, followed successively by F136 fish fed diet 40/20, and F120 fish fed diet 40/10, while the lowest *pparβ* expression was observed in F136 fish fed diet 40/10; however, there was no significant difference in *pparβ* expression between F136 fish fed diets 40/20 and 40/30, and between F136 fish fed diet 40/30 and F120 fish fed diet 40/10. The *ppargc1α* gene was up-regulated in the liver of F120 fish fed diet 40/20, whereas it was down-regulated in all the other treatment groups. The F120 fish fed diet 40/20 showed the highest *ppargc1α* expression, followed successively by the group comprising F120 fish fed diets 40/10 and 40/30, and F136 fish fed diet 40/20, while the lowest *ppargc1α* expression was observed in F136 fish fed diets 40/30; however, there was no significant difference in *ppargc1α* expression between F136 fish fed diets 40/10 and 40/30, and between F136 fish fed diets 40/10 and 40/30.

**Table 2:** Significant interactions between family and diet on nuclear gene expression in the liver of two juvenile rainbow trout *Oncorhynchus mykiss* families fed practical diets containing graded dietary lipid levels for 90 days ^1^.

Families ^2^	Practical Diets ^3^	Nuclear Gene Expression ^4^				
		*ucp2α*	*ucp2β*	*pparα*	*pparβ*	*pgc-1α*
**Individual treatment means**						
F120	40/10	0.000 ^d^	0.000 ^e^	0.000 ^e^	0.000 ^c^	0.000 ^b^
F120	40/20	0.912 ^a^	0.927 ^a^	0.527 ^b^	0.811 ^a^	0.336 ^a^
F120	40/30	0.477 ^b^	0.319 ^d^	0.589 ^a^	0.722 ^a^	−0.124 ^b^
F136	40/10	0.371 ^c^	0.612 ^b^	0.282 ^c^	−0.198 ^d^	−0.416 ^cd^
F136	40/20	0.585 ^b^	0.478 ^c^	0.159 ^d^	0.177 ^b^	−0.339 ^c^
F136	40/30	0.304 ^c^	0.304 ^d^	0.192 ^d^	0.106 ^bc^	−0.533 ^d^
**Pooled SEM**		0.058	0.032	0.019	0.426	0.042
**Main effect means**						
F120		0.463	0.415	0.372	0.511	0.071
F136		0.420	0.464	0.213	0.028	−0.429
	40/10	0.185	0.306	0.141	−0.099	−0.208
	40/20	0.748	0.702	0.340	0.494	−0.001
	40/30	0.390	0.312	0.393	0.414	−0.328
**ANOVA: *p* values**						
**Family (F)**		0.3846	0.0888	<0.0001	<0.0001	<0.0001
***F*-value/df ^5^**		0.81/1	3.43/1	102.48/1	193.38/1	212.41/1
**Diet (D)**		<0.0001	<0.0001	<0.0001	<0.0001	<0.0001
***F*-value/df ^5^**		47.85/2	98.92/2	96.77/2	114.71/2	30.95/2
**Interaction (FxD)**		0.0002	<0.0001	<0.0001	0.0003	0.0124
***F*-value/df ^5^**		19.87/2	135.50/2	197.48/2	16.78/2	6.48/2

^1^ Means represent average values of three tanks. The LSD procedure was applied on individual means because the two-factor interaction was significant. Individual treatment means within a column followed by different superscript letters were significantly different (*p* < 0.05); ^2^ Families of fish selected experimentally for low feed efficiency (F120) and high feed efficiency (F136) by USDA-ARS National Center of Cool and Coldwater Aquaculture (NCCCWA) in Leetown, WV, USA; ^3^ Diets formulated to contain 40% protein and 10% fat (40/10), 40% protein and 20% fat (40/20) or 40% protein and 30% fat (40/30); ^4^ Gene expression (*ucp2α*, *ucp2β*, *pparα*, *pparβ* and *pgc-1α*) values are presented as fold-change (log RQ) compared to the calibrator, which is the low feed efficiency family fed diet with lowest dietary fat level (family 120 fed diet 40/10); *ucp2α* refers to uncoupling protein 2 alpha gene, *ucp2β* to uncoupling protein 2 beta gene, *pparα* to peroxisome proliferator-activated receptor alpha gene, *pparβ* to peroxisome proliferator-activated receptor beta gene, and *pgc-1α* to peroxisome proliferators-activated receptor coactivator gene; and ^5^ *F*-values/degree of freedom (df) for family, diet and interaction, respectively.

### 2.2. Gene Expression in the Intestine

#### 2.2.1. Mitochondrial Genes in the Intestine

The effect of the interaction diet × family was highly significant (*p* < 0.01) on the expression of mitochondrial *nd1*, *cytb*, *cox1*, *cox2* and *atp6* genes in fish intestine (Table 3). The *nd1* gene was down-regulated in the intestine of F120 fish fed diet 40/20 and F136 fish fed diet 40/10, whereas it was up-regulated in all the other treatment groups. The F136 fish fed diet 40/30 showed the highest *nd1* expression, followed successively by the group comprising F120 fish fed diets 40/10 and 40/30 and F136 fish fed diet 40/20, and the F120 fish fed diet 40/20, whereas the lowest *nd1* expression was observed in F136 fish fed diet 40/10. The *cytb* gene was down-regulated in the in the intestine of F136 fish fed diet 40/30, whereas it was up-regulated in all the other treatment groups. The F136 fish fed diet 40/30 showed the highest *cytb* expression, followed successively by the group comprising F136 fish fed diet 40/20 and F120 fish fed diet 40/10, F120 fish fed diet 40/30, and F120 fish fed diet 40/20, while the lowest *cytb* expression was observed in F136 fish fed diet 40/10. The *cox1* gene was up-regulated in the intestine of F136 fish fed diet 40/30, whereas it was down-regulated in all the other treatment groups. The F136 fish fed diet 40/30 showed the highest *cox1* expression, followed successively by F120 fish fed diets 40/10, F136 fish fed diet 40/10, F120 fish fed diet 40/30, and F120 fish fed diet 40/20, while the lowest *cox1* expression was observed in F136 fish fed diet 40/20. The *cox2* gene was up-regulated in the intestine of F136 fish fed diet 40/30, whereas it was down-regulated in all the other treatment groups. The F136 fish fed diet 40/30 and the F120 fish fed diet 40/30 showed the highest and the lowest *cox2* expression, respectively, whereas the group comprising all the other treatments showed the intermediary expression. The *atp6* gene was down-regulated in the intestine of F136 fish fed diets 40/10 and 40/20, whereas it was up-regulated in all the other treatment groups. The group comprising F120 fish fed diet 40/30 and F136 fish fed diet 40/30 showed the highest *atp6* expression, followed successively by F120 fish fed diet 40/20, and the group comprising F120 fish fed diet 40/10 and F136 fish fed diet 40/20, while the lowest *atp6* expression was observed in F136 fish fed diets 40/10.

#### 2.2.2. Nuclear Genes in the Intestine

The effect of the interaction diet × family was highly significant (*p* < 0.01) on the expression of nuclear *ucp2α*, *ucp2β*, *pparα* and *pparβ* genes, and was significant (*p* < 0.05) on *ppargc1α* gene in fish intestine (Table 4). The *ucp2α* gene was up-regulated in the intestine of F136 fish fed diets 43/20 and 43/30, whereas it was down-regulated in all the other treatment groups. The F136 fish fed diet 40/20 showed the highest *ucp2α* expression, followed successively by the group comprising F136 fish fed diet 40/30, F120 fish fed diet 40/10, the group of F120 fish fed diets 40/20 and 40/30, whereas the lowest *ucp2α* expression was observed in F136 fish fed diet 40/10. The *ucp2β* gene was down-regulated in the intestine of F136 fish fed diet 40/10, whereas it was up-regulated in all the other treatment groups. The F136 fish fed diet 40/20 showed the highest *ucp2β* expression, followed successively by F136 fish fed diet 40/30, F120 fish fed diet 40/20, and F120 fish fed diet 40/30, while the lowest *ucp2β* expression was observed in the group comprising F120 fish fed diet 40/10 and F136 fish fed diet 40/10. The *pparα* gene was up-regulated in the intestine of all F120 fish fed diet 40/20 and F136 fish fed diet 40/30, whereas it was down-regulated in all the other treatment groups. The F136 fish fed diet 40/30 showed the highest *pparα* expression, followed successively by F120 fish fed diet 40/20, F120 fish fed diet 40/10, and the group comprising F120 fish fed diet 40/30 and F136 fish fed diet 40/20, while the lowest *pparα* expression was observed in F136 fish fed diet 40/10. The *pparβ* gene was down-regulated in the intestine of F136 fish fed diet 40/10, whereas it was up-regulated in all the other treatment groups. The group of fish comprising F120 fish fed diet 40/30 and F136 fish fed diet 40/30 showed the highest *pparβ* expression, followed successively by F120 fish fed diet 40/20, and F120 fish fed diet 40/10, while the lowest *pparβ* expression was observed in F136 fish fed diet 40/10; however, there was no significant difference in *pparβ* expression between the group of fish comprising F120 fish fed diet 40/30 and the F136 fish fed diets 40/20 and 40/30, and between F120 fish fed diets 40/20 and F136 fish fed diet 40/20. The *ppargc1α* gene was up-regulated in the intestine of F136 fish fed diets 40/20 and 40/30, whereas it was down-regulated in all the other treatment groups. The F136 fish fed diet 40/30 showed the highest *ppargc1α* expression, followed successively by the group comprising F136 fish fed diet 40/20, F120 fish fed diet 40/10, and F120 fish fed diet 40/20, while the lowest *ppargc1α* expression was observed in the group comprising F120 fish fed diet 40/30 and F136 fish fed diet 40/10.

**Table 3:** Significant interactions between family and diet on mitochondrial gene expression in the intestine of two juvenile rainbow trout *Oncorhynchus mykiss* families fed practical diets containing graded dietary lipid levels for 90 days ^1^.

Families ^2^	Practical Diets ^3^	Mitochondrial Gene Expression ^4^				
		Complex I: *nd1*	Complex III: *cytb*	Complex IV: *cox1*	Complex IV: *cox2*	Complex V: *atp6*
**Individual treatment means**						
F120	40/10	0.000 ^b^	0.000 ^b^	0.000 ^b^	0.000 ^b^	0.000 ^c^
F120	40/20	−0.206 ^c^	0.001 ^d^	−0.348 ^e^	−0.454 ^b^	0.186 ^b^
F120	40/30	0.415 ^b^	0.279 ^c^	−0.278 ^d^	−0.690 ^c^	0.556 ^a^
F136	40/10	−0.387 ^d^	−0.669 ^e^	−0.203 ^c^	−0.525 ^b^	−0.446 ^d^
F136	40/20	0.544 ^b^	0.370 ^b^	−0.620 ^f^	−0.482 ^b^	−0.085 ^c^
F136	40/30	0.757 ^a^	0.503 ^a^	0.404 ^a^	0.941 ^a^	0.591 ^a^
**Pooled SEM**		0.063	0.021	0.020	0.024	0.016
**Main effect means**						
F120		0.070	0.093	−0.208	−0.381	0.247
F136		0.305	0.068	−0.140	−0.022	0.020
	40/10	−0.194	−0.334	−0.102	−0.263	−0.223
	40/20	0.169	0.185	0.484	−0.468	0.050
	40/30	0.586	0.391	0.063	0.125	0.574
**ANOVA: *p* values**						
**Family (F)**		0.0007	0.1673	0.0011	<0.0001	<0.0001
***F*-value/df ^5^**		20.68/1	2.16/1	18.01/1	330.04/1	308.61/1
**Diet (D)**		<0.0001	<0.0001	<0.0001	<0.0001	<0.0001
***F*-value/df ^5^**		76.26/2	649.37/2	406.07/2	309.18/2	1305.84/2
**Interaction (FxD)**		<0.0001	<0.0001	<0.0001	<0.0001	<0.0001
***F*-value/df ^5^**		41.54/2	367.00/2	363.47/2	1086.27/2	117.93/2

^1^ Means represent average values of three tanks. The LSD procedure was applied on individual means because the two-factor interaction was significant. Individual treatment means within a column followed by different superscript letters were significantly different (*p* < 0.05); ^2^ Families of fish selected experimentally for low feed efficiency (F120) and high feed efficiency (F136) by USDA-ARS National Center of Cool and Coldwater Aquaculture (NCCCWA) in Leetown, WV, USA; ^3^ Diets formulated to contain 40% protein and 10% fat (40/10), 40% protein and 20% fat (40/20) or 40% protein and 30% fat (40/30); ^4^ Gene expression (*nd1*, *cytb*, *cox1*, *cox2* and *atp6*) values are presented as fold-change (log RQ) compared to the calibrator, which is the low feed efficiency family fed diet with lowest dietary fat level (family 120 fed diet 40/10); and ^5^ *F*-values/degree of freedom (df) for family, diet and interaction, respectively.

**Table 4:** Significant interactions between family and diet on nuclear gene expression in the intestine of two juvenile rainbow trout *Oncorhynchus mykiss* families fed practical diets containing graded dietary lipid levels for 90 days ^1^.

Families ^2^	Practical Diets ^3^	Nuclear Gene Expression ^4^				
		*ucp2α*	*ucp2β*	*pparα*	*pparβ*	*pgc-1α*
**Individual treatment means**						
F120	40/10	0.000 ^c^	0.000 ^e^	0.000 ^c^	0.000 ^c^	0.000 ^c^
F120	40/20	−0.219 ^d^	0.364	0.349 ^b^	0.681 ^b^	−0.313 ^d^
F120	40/30	−0.305 ^d^	0.231 ^d^	−0.272 ^d^	0.894 ^a^	−0.692 ^e^
F136	40/10	−0.618 ^e^	−0.054 ^e^	−0.537 ^e^	−0.495 ^d^	−0.610 ^e^
F136	40/20	0.647 ^a^	0.915 ^a^	−0.150 ^d^	0.824 ^ab^	0.504 ^b^
F136	40/30	0.475 ^b^	0.668 ^b^	0.783 ^a^	0.940 ^a^	0.913 ^a^
**Pooled SEM**		0.018	0.036	0.046	0.062	0.088
**Main effect means**						
F120		−0.175	0.198	0.025	0.525	−0.335
F136		0.168	0.510	0.032	0.423	0.269
	40/10	−0.309	−0.027	−0.268	−0.248	−0.305
	40/20	0.214	0.639	0.100	0.752	0.095
	40/30	0.085	0.450	0.255	0.917	0.111
**ANOVA: *p* values**						
**Family (F)**		<0.0001	<0.0001	0.8623	0.0672	<0.0001
***F*-value/df ^5^**		529.63/1	109.98/1	0.03/1	4.05/1	70.23/1
**Diet (D)**		<0.0001	<0.0001	<0.0001	<0.0001	0.0007
***F*-value/df ^5^**		446.07/2	178.01/2	67.57/2	204.00/2	14.27/2
**Interaction (FxD)**		<0.0001	<0.0001	<0.0001	<0.0005	<0.0001
***F*-value/df ^5^**		1042.60/2	39.13/2	192.78/2	15.14/2	80.92/2

^1^ Means represent average values of three tanks. The LSD procedure was applied on individual means because the two-factor interaction was significant. Individual treatment means within a column followed by different superscript letters were significantly different (*p* < 0.05); ^2^ Families of fish selected experimentally for low feed efficiency (F120) and high feed efficiency (F136) by USDA-ARS National Center of Cool and Coldwater Aquaculture (NCCCWA) in Leetown, WV, USA; ^3^ Diets formulated to contain 40% protein and 10% fat (40/10), 40% protein and 20% fat (40/20) or 40% protein and 30% fat (40/30); ^4^ Gene expression (*ucp2α*, *ucp2β*, *pparα*, *pparβ* and *pgc-1α*) values are presented as fold-change (log RQ) compared to the calibrator, which is the low feed efficiency family fed diet with lowest dietary fat level (family 120 fed diet 40/10); *ucp2α* refers to uncoupling protein 2 alpha gene, *ucp2β* to uncoupling protein 2 beta gene, *pparα* to peroxisome proliferator-activated receptor alpha gene, *pparβ* to peroxisome proliferator-activated receptor beta gene, and *pgc-1α* to peroxisome proliferators-activated receptor coactivator gene; and ^5^ *F*-values/degree of freedom (df) for family, diet and interaction, respectively.

### 2.3. Gene Expression in the Muscle

#### 2.3.1. Mitochondrial Genes in the Muscle

The effect of the interaction diet × family was highly significant (*p* < 0.01) on the expression of mitochondrial *nd1*, *cytb*, *cox1*, *cox2* and *atp6* genes in fish muscle (Table 5). The *nd1* gene was down-regulated in the muscle of the group comprising F136 fish fed diet 40/10 and 40/30, whereas it was up-regulated in all the other treatment groups. The group comprising F120 fish fed diets 40/20 and F136 fish fed diet 40/20 showed the highest *nd1* expression, followed successively by F120 fish fed diets 40/30 and F120 fish fed diet 40/10, whereas the lowest *nd1* expression was observed in F136 fish fed diet 40/30. The *cytb* gene was up-regulated in the in the muscle of all the treatment groups. The *cytb* expression was higher in the group comprising F136 fish fed diets 40/10 and 40/20 than F120 fish fed diet 40/30, which was higher than that observed in F120 fish fed diet 40/10; however, there was no significant difference in *cytb* expression between F120 fish fed diet 40/20 and all the F136 fish groups, and between F120 fish fed diets 40/20 and 40/30. The *cox1* gene was up-regulated in the muscle of all the treatment groups. The group of fish comprising F120 fed diets 40/20 and 40/30 showed the highest *cox1* expression, followed successively by F136 fish fed diets 40/20, F136 fish fed diets 40/30, and F136 fish fed diet 40/10, while the lowest *cox1* expression was observed in F120 fish fed diet 40/10. The *cox2* gene was down-regulated in the muscle of F136 fish fed diet 40/30, whereas it was up-regulated in all the other treatment groups. The F120 fish fed diet 40/30 showed the highest and the lowest *cox2* expression, followed successively by the group comprising F120 fish fed diet 40/30 and F136 fish fed diets 40/10 and 40/20, and F120 fish fed diet 40/10, whereas the lowest *cox2* expression was observed in F136 fish fed diet 40/30. The *atp6* gene was down-regulated in the muscle of F136 fish fed diet 40/30, whereas it was up-regulated in all the other treatment groups. The group comprising F120 fish fed diets 40/20 and 40/30 and F136 fish fed diets 40/20 and 40/30 showed the highest *atp6* expression, whereas the lowest *atp6* expression was observed in the group comprising F120 fish fed diet 40/10 and F136 fish fed diets 40/30.

**Table 5:** Significant interactions between family and diet on mitochondrial gene expression in the muscle of two juvenile rainbow trout *Oncorhynchus mykiss* families fed practical diets containing graded dietary lipid levels for 90 days ^1^.

Families ^2^	Practical Diets ^3^	Mitochondrial Gene Expression ^4^				
		Complex I: *nd1*	Complex III: *cytb*	Complex IV: *cox1*	Complex IV: *cox2*	Complex V: *atp6*
**Individual treatment means**						
F120	40/10	0.000 ^c^	0.000 ^c^	0.000 ^e^	0.000 ^d^	0.000 ^b^
F120	40/20	0.638 ^a^	0.598 ^ab^	0.614 ^a^	0.510 ^b^	0.506 ^a^
F120	40/30	0.401 ^b^	0.540 ^b^	0.589 ^a^	0.908 ^a^	0.475 ^a^
F136	40/10	−0.563 ^e^	0.691 ^a^	0.151 ^d^	0.689 ^b^	0.446 ^a^
F136	40/20	0.620 ^a^	0.685 ^a^	0.373 ^b^	0.720 ^b^	0.370 ^a^
F136	40/30	−0.262 ^d^	0.650 ^ab^	0.246 ^c^	−0.327 ^e^	−0.176 ^b^
**Pooled SEM**		0.026	0.045	0.026	0.048	0.062
**Main effect means**						
F120		0.346	0.379	0.401	0.473	0.227
F136		−0.068	0.676	0.257	0.361	0.213
	40/10	−0.281	0.346	0.076	0.345	0.223
	40/20	0.629	0.642	0.494	0.615	0.438
	40/30	0.070	0.595	0.418	0.291	0.150
**ANOVA: *p* values**						
**Family (F)**		<0.0001	<0.0001	<0.0001	0.0154	0.0439
***F*-value/df ^5^**		389.97/1	63.70/1	47.14/1	7.96/1	5.07/1
**Diet (D)**		<0.0001	<0.0001	<0.0001	<0.0001	0.0015
***F*-value/df ^5^**		638.31/2	24.52/2	149.77/2	25.76/2	11.67/2
**Interaction (FxD)**		<0.0001	<0.0001	<0.0001	<0.0001	<0.0001
***F*-value/df ^5^**		91.30/2	28.30/2	51.38/2	213.61/2	39.27/2

^1^ Means represent average values of three tanks. The LSD procedure was applied on individual means because the two-factor interaction was significant. Individual treatment means within a column followed by different superscript letters were significantly different (*p* < 0.05); ^2^ Families of fish selected experimentally for low feed efficiency (F120) and high feed efficiency (F136) by USDA-ARS National Center of Cool and Coldwater Aquaculture (NCCCWA) in Leetown, WV, USA; ^3^ Diets formulated to contain 40% protein and 10% fat (40/10), 40% protein and 20% fat (40/20) or 40% protein and 30% fat (40/30); ^4^ Gene expression (*nd1*, *cytb*, *cox1*, *cox2* and *atp6*) values are presented as fold-change (log RQ) compared to the calibrator, which is the low feed efficiency family fed diet with lowest dietary fat level (family 120 fed diet 40/10); and ^5^ *F*-values/degree of freedom (df) for family, diet and interaction, respectively.

#### 2.3.2. Nuclear Genes in the Muscle

The effect of the interaction diet × family was highly significant (*p* < 0.01) on the expression of nuclear *ucp2α*, *ucp2β*, *pparα* and *ppargc1α* genes, whereas the effects of the diet and the family were significant (*p* < 0.05) on *pparβ* gene in fish muscle (Table 6). The *ucp2α* gene was down-regulated in the muscle of all the treatment groups. The F120 fish fed diet 40/10 showed the highest *ucp2α* expression, followed successively by F136 fish fed diet 40/30, and the group comprising F120 fish fed diets 40/20 and 40/30, whereas the lowest *ucp2α* expression was observed in the group comprising F136 fish fed diets 40/10 and 40/20. The *ucp2β* gene was down-regulated in the muscle of F136 fish fed diet 40/10, whereas it was up-regulated in all the other treatment groups. The F120 fish fed diet 40/30 showed the highest *ucp2β* expression, followed successively by the group comprising F120 fish fed diet 40/20 and F120 fish fed diet 40/10, and F136 fish fed diet 40/20, while the lowest *ucp2β* expression was observed in the group comprising F136 fish fed diet 40/10. The *pparα* gene was down-regulated in the muscle of all F120 fish fed diet 40/20, whereas it was up-regulated in all the other treatment groups. The F136 fish fed diet 40/20 showed the highest *pparα* expression, followed successively by the group comprising F136 fish fed diet 40/10 and 40/30, F120 fish fed diet 40/30, and the F120 fish fed diet 40/10, while the lowest *pparα* expression was observed in F120 fish fed diet 40/20. The gene *pparβ* was up-regulated in the muscle of all the treatment groups. The F136 fish family (LFE) showed a higher *pparβ* expression than the F120 fish family (HFE). The fish fed the diets 40/20 and 40/30 showed similar *pparβ* expressions, which were higher than those observed in fish fed the diet 40/10. The *ppargc1a* gene was up-regulated in the muscle of all the treatment groups. The F120 fish fed diet 40/10 showed a significantly low *ppargc1a* expression compared to all the other treatment groups, which had *ppargc1a* expression levels that were not significantly different.

**Table 6:** Significant interactions between family and diet on nuclear gene expression in the muscle of two juvenile rainbow trout *Oncorhynchus mykiss* families fed practical diets containing graded dietary lipid levels for 90 days ^1^.

Families ^2^	Practical Diets ^3^	Nuclear gene expression ^4^				
		*ucp2α*	*ucp2β*	*pparα*	*pparβ*	*pgc-1α*
**Individual treatment means**						
F120	40/10	0.000 ^a^	0.000 ^c^	0.000 ^d^	0.000	0.000 ^b^
F120	40/20	−0.798 ^c^	0.500 ^b^	−0.349 ^e^	0.227	0.324 ^a^
F120	40/30	−0.854 ^c^	0.761 ^a^	0.153 ^c^	0.186	0.330 ^a^
F136	40/10	−0.926 ^d^	−0.435 ^e^	0.250 ^b^	0.280	0.422 ^a^
F136	40/20	−0.929 ^d^	0.271 ^d^	0.741 ^a^	0.315	0.367 ^a^
F136	40/30	−0.661 ^b^	0.405 ^b^	0.326 ^b^	0.324	0.288 ^a^
**Pooled SEM**		0.037	0.048	0.051	0.045	0.062
**Main effect means**						
F120		−0.551	0.422	−0.110	0.137 ^b^	0.218
F136		−0.839	−0.100	0.439	0.306 ^a^	0.359
	40/10	−0.463	−0.217	0.125	0.140 ^b^	0.211
	40/20	−0.864	0.117	0.117	0.271 ^a^	0.345
	40/30	−0.757	0.583	0.340	0.255 ^a^	0.308
**ANOVA: *p* values**						
**Family (F)**		<0.0001	<0.0001	<0.0001	0.0006	0.0168
***F*-value/df ^5^**		92.04/1	180.20/1	175.74/1	21.10/1	7.70/1
**Diet (D)**		<0.0001	<0.0001	0.0606	0.0256	0.1241
***F*-value/df ^5^**		63.61/2	142.96/2	3.57/2	5.05/2	2.50/2
**Interaction (FxD)**		<0.0001	0.0010	<0.0001	0.1304	0.0065
***F*-value/df ^5^**		122.49/2	10.99/2	67.98/2	2.43/2	7.88/2

^1^ Means represent average values of three tanks. The LSD procedure was applied on individual means because the two-factor interaction was significant. Individual treatment means within a column followed by different superscript letters were significantly different (*p* < 0.05); ^2^ Families of fish selected experimentally for low feed efficiency (F120) and high feed efficiency (F136) by USDA-ARS National Center of Cool and Coldwater Aquaculture (NCCCWA) in Leetown, WV, USA; ^3^ Diets formulated to contain 40% protein and 10% fat (40/10), 40% protein and 20% fat (40/20) or 40% protein and 30% fat (40/30); ^4^ Gene expression (*ucp2α*, *ucp2β*, *pparα*, *pparβ* and *pgc-1α*) values are presented as fold-change (log RQ) compared to the calibrator, which is the low feed efficiency family fed diet with lowest dietary fat level (family 120 fed diet 40/10); *ucp2α* refers to uncoupling protein 2 alpha gene, *ucp2β* to uncoupling protein 2 beta gene, *pparα* to peroxisome proliferator-activated receptor alpha gene, *pparβ* to peroxisome proliferator-activated receptor beta gene, and *pgc-1α* to peroxisome proliferators-activated receptor coactivator gene; and ^5^ *F*-values/degree of freedom (df) for family, diet and interaction, respectively.

## 3. Discussion

The present experiment aimed at studying the effects of graded dietary lipid levels on the expression of nuclear and mitochondrial genes involved in the mitochondrial electron transport chain (ETC) in low- and high feed efficient (FE) families of rainbow trout. The designation of the experimental fish as high-FE (F136) and low-FE (F120) was not reflected in the results based on the weight gain and feed efficiency (FE) [25]. The fish designated low-FE had better growth rate and higher FE and the evaluation of the results was based on the differences in growth rate and FE obtained from the present study and not based on the designation by the breeding program. The discrepancy between the assigned phenotypic feed efficiency and the results obtained could be due to low heritability values for feed efficiency observed in rainbow trout and other salmonids that ranges from 3% ± 10% [26] and 6% ± 10% [27]. The results showed that there is an interactive effect of the dietary lipid levels and the phenotypic feed efficiency (growth rate and FE) as observed in the present study on the expression of the mitochondrial genes *nd1*, *cytb*, *cox1*, *cox2* and *atp6* and nuclear genes *ucp2α*, *ucp2β*, *pparα*, *pparβ* and *ppargc1α* in fish liver, intestine and muscle, except on *ppargc1α* in the muscle which was affected by the diet and the family separately. Also, the results revealed that the expression of mitochondrial genes is associated with that of nuclear genes involved in ETC in fish liver, intestine and muscle. Furthermore, this work showed that the expression of mitochondrial genes parallels with the expression of genes encoding uncoupling proteins (UCP) in the liver and the intestine of rainbow trout. The significant family × diet interaction suggests that the two factors are important and necessary in explaining mitochondrial function and other associated growth performance characteristic responses. This implies that the fast growing fish with improved nutrient retention associated with up/down regulation of mitochondrial and nuclear genes fed 40/20 diet might not necessarily show the same responses when offered a different diet.

In humans, the molecular basis of mitochondrial dynamic and energetics has extensively been studied, in order to cure and/or prevent food-related pathologies [20]. In farmed animals such as the broiler chicken, the study of the mitochondrial dynamics in relation to feed utilization started in 2002 and has spurred scientific interest ever since, which substantially contributed to establishing the basic information in order to understand the relationship between mitochondrial function and feed efficiency in farmed animals such as poultry and livestock species [6]. In fish, although several studies have been conducted on dietary energy flow at the organismal level [28,29,30], little attention has been focused on the molecular mechanisms underlying the efficiency of energy flow regulation at the mitochondrial level [16,17,25]. Furthermore, in the present study the interpretation of results of the expression of individual genes involved in ETC, in response to the diet, the fish family and their interaction, is complex. Nevertheless, the difference in the expression of these genes in the different tissues analyzed is likely due to the high tissue-specific diversity in mitochondrial shape, organization and functioning, which are related to differences in energy demand and supply and also tissue-specific regulation of mitochondrial fission and fusion [20]. The highest expression of *nd1*, *cytb* and *cox1* was observed in the liver and the muscles of fish with high growth rate (F120 family) fed 20% dietary lipid. This high gene expression level is associated with the high growth rate with the same diet in a nutritional study conducted concomitantly with this study [25]. This result confirms the previous finding that 18%–20% dietary lipids are adequate for maximum growth and feed utilization in rainbow trout [21,31]. The fact that the highest expression of the mitochondrial genes was observed in the intestine of fish designated as high-FE fish (F136 family) but with unexpected low feed efficiency data (unpublished data) when fed diet containing 30% dietary lipid was in contrast with previous work (21) which presented a down-regulation of these genes in rainbow trout fed a diet with the same dietary lipid level and 42% crude protein. Excess dietary lipid appears to increase gene expression in the intestine of high-FE (slower growing) fish with worst feed efficiency contrary to our expectation that fish designated high-FE should have better growth which was not the case in this instance. This high up-regulation of gene expression by excess fat in high-FE fish is in agreement with the findings of Nakamura *et al.* [22] who reported that dietary lipid regulates the expression of genes that are involved in energy and lipid metabolism in mammals, through the action of transcriptional factors such as sterol regulatory element binding protein-1c (Srebp-1c) and peroxisome proliferator-activated receptor α (PPARα). Furthermore, Schothorst and colleagues [32] observed that dietary intake of eicosapentaenoic (EPA) and docosahexaenoic (DHA) induced dose–dependent changes in the mouse small intestine gene expression and suggested that increased catabolism of lipids leads to upregualtion of especially genes involved in lipid catabolism. It should be noted that small intestine mediates the entry of nutritional lipids and is one of the main sites of β-oxidation [33] and the intestinal overload of fat on the 40/30 diet concomitantly lead to the increased number of the differentially expressed genes. Because in the previous study the dietary protein level was 42%, whereas in the present study it was 40%, it is likely that this discrepancy in mitochondrial gene expression is related to the dietary protein level interacting with dietary lipid, with the lower dietary protein level producing a higher response in the expression of mitochondrial genes involved in energy production in the cells. However, with the same dietary lipid level, there is down-regulation of mitochondrial genes by 40% dietary protein, compared to 42% dietary protein in the previous study and could be due to the fact higher protein level induces a number of energy sensing, producing and utilizing pathways, especially genes associated with oxidative phosphorylation (OXPHOS). This is supported by the findings Oster and colleagues [34] that genes associated with OXPHOS, biosynthesis of steroids, and valine, leucine and isoleucine degradation were diminished in low protein diets. Additionally, from the results of the current experiment, it may be postulated that in the low-FE fish, the supply of excess dietary lipid might induce the expression of genes encoding enzymes (complexes) involved in the lipid metabolism in the intestine, in order to maximize energy production from digested lipid, as an adaptation feature to compensate for the low-FE phenotype. Moreover, the observation of higher mitochondrial gene expressions in the liver and muscle of F120 fish fed diet 40/20 than those observed in the intestine of F136 fish fed diet 40/20 suggests that fish with fast growth rate may be well correlated with the expression of the mitochondrial genes in the liver and the muscle. Consistently, the expression of the genes encoding the nuclear proteins involved in ETC and lipid metabolism, such as PPAR and PPARGC-1α [22], is highest in the liver of fish with better growth rate (F120) fed diet 40/20, whereas it is higher in the intestine of slower growth rate (F136 family) fed diet containing 30% dietary lipid. Thus, the high expression of mitochondrial genes in low-FE fish fed diet 40/30 is associated with the high expression of nuclear genes. The PPAR and PPARGC-1α are nuclear proteins that are known to induce nuclear respiratory factor-1 and mitochondrial transcription factor A in mammals [35]. Also, PPAR is a transcription factors of the nuclear receptor family that is required for the induction of the expression of genes involved in mitochondrial and peroxisomal β-oxidation in mammals [22,36,37,38]. It is well-established that the PPAR regulate fatty acid oxidation in rodent and this is suggested to occur also in non-rodent animals [22], such as fish. On the other hand, the high expression of mitochondrial genes in the present study tends to be coupled with the expression of uncoupling proteins (UCP) in the liver and the intestine, but not in the muscle. This result is in contrast with that obtained with chicken which showed higher avian UCP mRNA expression in breast muscles than in the other tissues [39]. The association of the expression of genes involved in ETC with that of UCP genes confirms the important role of UCP in mitochondrial function, as previous observations have showed that uncoupling reduces reactive oxygen species (ROS) production in drosophila *Drosophila melanogaster* [40] and lowers ROS and lipid oxidative damage in frog *Rana temporaria* tadpoles [41]. The ROS production is responsible of oxidative damage in cells and at a certain level induces mitochondrial dysfunction [6,32].

## 4. Materials and Methods

The Present study was approved by the West Virginia State University Institutional Review Board (WVSU IRB# 11-2004), which determined that the methodology used in this experiment were in accordance with the guidelines for care and use of laboratory animals established by the Institute of Laboratory Animal Resources Commission (ILAR) and the guidelines for use of fishes in research set forth by the Uses of Fishes in Research Committee (UFRC). This study was conducted concomitantly with a nutritional study investigating the relation between growth, FE and mitochondrial enzyme activities in rainbow trout fed a crude protein level fixed at 40%, and graded levels of dietary lipids [25]. The fish rearing protocol was similar in both studies. Accordingly, only a brief description of the rearing conditions will be presented in this section, while particular emphasis will be given to the materials and methods used to study effect of dietary lipid on gene expression. The present study was approved by the West Virginia State University Institutional Review Board (WVSU IRB#11-2004), which determined that the methodology used in this experiment were in accordance with the guidelines for care and use of laboratory animals established by the Institute of Laboratory Animal Resources Commission (ILAR) and the guidelines for use of fishes in research set forth by the Uses of Fishes in Research Committee [42].

### 4.1. Fish Husbandry and Feeding

Two different families of juvenile all-female rainbow trout, of the same age, designated F120 for high-FE and F136 for low-FE were obtained from the USDA-ARS National Center of Cool and Coldwater Aquaculture (NCCCWA) in Leetown, West Virginia, USA. The families were generated from sires and dams that were evaluated for feed efficiency under laboratory conditions as part of the NCCWA’s growth improvement lines, and the FE potential of these families was based on the FE and growth performances of their parents. Out of the 96 families with different FE potentials that were produced by the NCCCWA, the parents used to generate F136 (high-FE) family showed the highest average parental body weight at 10 months posthatch (579.2 g and average breeding value for the body weight at 10 months posthatch was 57.65 g), while the parents used to generate the F120 (low-FE) family had 375.5 g average parental body weight at 10 months post hatch and average breeding value of −62.09 g for the body weight at 10 months posthatch. However, the fish used in this study had not previously been evaluated to determine the effects of either genotype or dietary composition on mitochondrial and nuclear genes expression involved in oxidative phosphorylation. The fish were reared in an indoor flow-through system comprising glass tanks (152 L each) supplied with a continual flow of dechlorinated city water at a rate of 1.5 L·min^−1^. Water temperature was thermostatically controlled at 12 ± 2 °C and each tank was individually aerated to maintain a dissolved oxygen concentration above 6.5 mg·L^−1^. A 12 h light and 12 h dark photoperiod was simulated with electrically timed fluorescent lights in a temperature-controlled room. The fish were acclimated to the system for 14 days during which they were hand-fed one of the experimental diet containing 40% crude protein and 10% crude fat to satiation twice daily. Then, fish from the same family were randomly distributed in 9 tanks, with a density of 10 fish per tank, for a total of 18 tanks. The initial weight ± SD of F120 fish was 217.66 ± 2.24 g while that of F136 fish was 205.47 ± 1.27 g. Three experimental practical diets were formulated to contain 40% protein and 10% fat (diet 40/10), 40% protein and 20% fat (diet 40/20), and 40% protein and 30% fat (diet 40/30) (Table 7). The 40% protein level was chosen because it is similar to the lowest level usually used in commercial trout production, while the three fat levels were selected to produce low-, medium- and high nutrient-dense diets. Diets were isonitrogenous and dietary energy varied with dietary lipid level (Table 1). These diets were made using a Wenger X85 (Wengner Manufacturing, Inc., Sabetha, KS, USA) research scale extruder. All ingredients were supplied by Rangen (Buhl, ID, USA). Each diet was randomly assigned to triplicate tanks per family, in a 3 × 2 factorial experimental design (3 diets × 2 families × 3 replicates = 18 tanks or experimental units). During the experiment, fish were hand-fed to apparent satiation twice daily (7 days·week^−1^) at 08:00 and 16:00, for 90 days. Satiation feeding was achieved by allowing fish to eat until feeding activity stopped, with no feed remaining in the tank. Fish in each aquarium were fasted for 24 h prior to sampling. At the end of the 90-day feeding trial, fish in each aquarium were killed by an overdose of MS-222 (300 mg·L^−1^) and immediately put on ice.

**Table 7:** Feed formulations and proximate composition of experimental diets fed to two families of rainbow trout (*Oncorhynchus mykiss*) during 90 days.

Ingredient (g/100 g Diet, as-Fed Basis) ^2^	Experimental Diets ^1^		
Protein/lipid levels (%)	40/10	40/20	40/30
Menhaden fish meal (69% crude protein)	30.00	30.00	30.00
Soybean meal (47% crude protein)	15.00	15.00	15.00
Blood meal (88% crude protein)	3.00	5.00	6.00
Feather meal (84% crude protein)	5.00	5.00	5.00
Wheat flour (11% crude protein)	9.00	9.00	9.00
Brewe’s yeast (46% crude protein)	2.00	2.00	2.00
Wheat midds (15% crude protein)	28.61	16.25	4.75
Vitamin premix	0.40	0.40	0.40
Mineral premix	0.10	0.10	0.10
Stay-C	0.14	0.14	0.14
Choline chloride	0.58	0.58	0.58
Dicalcium Phosphate	0.4	0.4	0.4
Calcium propionate	0.13	0.13	0.13
Fish oil	5.60	16.00	26.50
Total	100.00	100.00	100.00
**Proximate composition**			
Crude protein	40.90	40.78	39.93
Fat	10.02	20.04	30.19
Moisture	7.85	5.29	7.03
Ash	8.45	7.58	7.55
Gross energy (kJ/g)	19.28	21.33	24.32

^1^ Diets formulated to contain 40% protein and 10% fat (40/10), 40% protein and 20% fat (40/20) or 40% protein and 30% fat (40/30); and ^2^ All ingredients were supplied by Rangen (Buh1, ID, USA).

### 4.2. Sample Collection

The liver, the intestine and the muscles are the primary tissues selected for mitochondrial and nuclear gene expression analysis in this study, because their oxidative capacity may reflect growth rates of fish; liver has a high metabolic capacity for oxidation and synthesis of numerous metabolites, the digestive tract determines the rates of nutrient assimilation, and the skeletal muscle has predominant role in the deposition of material during growth, locomotion and whole-body metabolic homeostasis. From three fish randomly chosen from each aquarium, a transverse slice of muscle (approximately 100 mg) located beneath the dorsal fin, the liver (~50–100 mg), and the intestine (~100 mg, proximal part of the intestine, just after the stomach) were collected for the analysis of the expression of genes involved in mitochondrial electron transport chain. Following collection, the liver, muscle and intestine tissues (50–100 mg) were placed in 5–10 volumes of RNAlater^®^ solution (Applied Biosystems/Ambion, Austin, TX, USA), and incubated overnight at 4 °C. The supernatant was discarded the following morning and samples stored at −80 °C until the RNA isolation step.

### 4.3. Gene Expression Analysis

#### 4.3.1. Extraction and Purification of Total RNA

The total RNA was extracted from the liver and intestine using RNAqueous-4PCR^®^ kit (Applied Biosystems/Ambion #AM1914) according to the manufacturer’s instructions, and the RNA was then stored at −80 °C. A larger volume of lysis buffer (15–20 volumes) was used during the isolation of RNA from the intestine (compared with that used with the liver sample, 10–12 volumes) and a clarifying spin was applied after the homogenization step in order to avoid the clogging of the filter. The isolation of RNA from the muscles was performed using a modification of the guanidinium thiocyanate-phenol-chloroform extraction method described by Chomczynski and Sacchi [43]. Briefly, approximately 50 mg of tissue was homogenized in 1 mL of TRI reagent (Sigma #T9424, St. Louis, MO, USA) using a tissue lyser (Qiagen Inc., Valencia, CA, USA) and incubated at room temperature for 5 min. Proteinase K, 1 µL (Qiagen #19131) was added to the sample, vortexed vigorously 15 s, and incubated at 55 °C in a water bath for 30 min. Pure chloroform, 200 μL was added under a laminar flow hood, vortexed vigorously for 15 s, then incubated at room temperature for 5 min while continually mixing on a Rocker II Model 260350 (Boekel Scientific, Feasterville, PA, USA). The tubes were then centrifuged at maximum speed for 20 min at 4 °C to separate the samples into three phases: a colorless upper aqueous phase (containing RNA), a milky interphase (DNA), and a lower, reddish-brown colored phase (proteins, *etc.*). The upper aqueous phase containing RNA was transferred to another microcentrifuge tube, 500 μL of isopropyl alcohol was added, and the samples incubated at room temperature for 10 min. The RNA was pelleted by centrifuging at maximum speed for 15 min at 4 °C, and the supernatant discarded. One ml of 80% ethanol was then added, vortexed for 15 s, and centrifuged again at maximum speed for 5 min at 4 °C. The supernatant from this spin was discarded and the tubes allowed to air dry at room temperature for 5 min inverted on a paper towel. The final pellet was then suspended in 100 µL of nuclease-free water and incubated at 55 °C in a water bath for 10 min to allow RNA to completely dissolve.

#### 4.3.2. DNase I Treatment and Quantification of Total RNA

All isolated samples were treated with DNase I in order to remove any carry-over contaminating genomic DNA using the reagents and protocol provided in the RNAqueous-4PCR^®^ kit. After inactivation of DNase I, the RNA was transferred to a 0.2 mL PCR tube and quantified with a ND-1000 Spectrophotometer (NanoDrop Products, Wilmington, DE, USA). For the liver and intestine samples, total RNA was measured using the elution solution provided in the RNA Aqueous-4PCR^®^ kit as a reference blank. For the muscle samples, total RNA was measured using nuclease-free water as the reference blank.

#### 4.3.3. cDNA Synthesis

For cDNA Synthesis, 2 µg of each total RNA sample (1 µg if less than 200 ng/µL concentration according to NanoDrop measurements) was converted to single-stranded cDNA according to the protocol for High-Capacity cDNA Reverse Transcription Kit (Applied Biosystems/Ambion #4368813). Samples were then removed from the thermocycler and stored at −20 °C until Real-Time PCR.

#### 4.3.4. Real-Time PCR Genes and Primers

In this work, the mitochondrial genes encoding the subunits of the respiratory chain that were studied comprised Complex I: NADH dehydrogenase subunit 1 (*nd1*); Complex III: Cytochrome b (*cytb*); Complex IV: Cytochrome c oxidase subunits 1 (*cox1*); Complex IV: Cytochrome c oxidase subunits 2 (*cox2*); Complex V: ATP synthase subunit 6 (*atp6*). The selected nuclear genes evaluated were peroxisome proliferators-activated receptor (*pparα* and *pparβ*); peroxisome proliferators-activated receptor coactivator (*ppargc1α*) and uncoupling proteins (*ucp2α* and *ucp2β*). Two reference housekeeping genes, the β-*actin* and the elongation factor 1-α (*ef1*α) were utilized to normalize the expression levels of the target genes. The β-*actin* and the *ef1*α were chosen as the reference genes based on the results of Olsvik and co-workers [44], who evaluated commonly used endogenous controls and found that these two genes displayed stable expression levels across different tissue types and life stages in Atlantic salmon *Salmo salar*, a close relative of rainbow trout. The primers (Table 8) for *nd1*, *cox1*, *atp6* and *β*-*actin* were identical to those described by O’Dowd and co-workers [45]. The primer for *cox2* was adapted from Hook and co-workers [46], and that for *ef1*α was adapted from Bobe and co-workers [47]. The primers for mt-*cytb*, *ucp2α*, *ucp2β*, *pparα*, *pparβ*, and *ppargc1α* were designed with Primer Express Software 3.0 (Applied Biosystems) using the highest conserved region from a multiple sequence alignment of rainbow trout expressed sequence tags (ESTs) with CLUSTALW online software. The primer pair with the lowest penalty scores was selected and custom ordered from Applied Biosytems (#4304971).

#### 4.3.5. Primer Optimization

A primer concentration optimization procedure was performed according to the manufacturer’s instructions for the Power Sybr^®^ Green Master Mix Protocol (Applied Biosystems #4367218C). An optimization procedure involving serial dilutions (10, 100 and 200 nM) of cDNA was also performed to determine the correct amount of template to include in each reaction. According to the results, 100 nM of the forward and reverse primers for each gene and 50 ng of cDNA were utilized.

**Table 8:** Target Genes and Primer Sequences used in mitochondrial gene expression analysis in two families of rainbow trout (*Oncorhynchus mykiss*) fed practical diets containing graded levels of dietary lipid for 90 days.

Genes	Origin	GenBank Accession No.	Primers	Sequence 5' to 3'
*β*-*actin*	nDNA	AJ438158	Forward	gaagatgaaatcgccgcactgg
			Reverse	ctttctggcccatcccaacca
*Elongation factor 1-α*	nDNA	AF498320	Forward	agcgcaatcagcctgagaggta
			Reverse	gctggacaagctgaaggctgag
*NADH dehydrogenase subunit 1*	mtDNA	NP_008290	Forward	tagcatacattgtacccgttctgttagcag
			Reverse	aatagttttaggccgtctgcgatgg
*Cytochrome c oxidase I*	mtDNA	NP_008292	Forward	ctcaaccaaccacaaagacattggc
			Reverse	tcacgttatagatttggtcatccccc
*Cytochrome c oxidase II*	mtDNA	NP_008293	Forward	gaggcaataaaggctgtttggt
			Reverse	gaggccgttccttctttaggtgtaa
*Cytochrome b*	mtDNA	NC_001717	Forward	tggccaacctccgaaaaac
			Reverse	ggaggtcgactagtgcgtcatt
*ATPase subunit 6*	mtDNA	NP_008295	Forward	cttcttcgaccaatttatgagcccc
			Reverse	Tcggttgatgaaccacccttgc
*Uncoupling protein 2 alpha*	nDNA	NM_001124654.1	Forward	tccatgcctgcacgaattt
			Reverse	tttagcagatgccccaagtga
*Uncoupling protein 2 beta*	nDNA	NM_001124571.1	Forward	ggaaaaggtgcggcagcta
			Reverse	accaaacacaccccgatacc
*Peroxisome proliferator-activated receptor alpha*	nDNA	NM_001197211.1	Forward	gctggagctggatgacagtga
			Reverse	gcggtctccacagcagat
*Peroxisome proliferator-activated receptor beta*	nDNA	NM_001197207.1	Forward	cctggcgggagagaaagc
			Reverse	cagggatttgagatccgagcta
*Proliferator-activated receptor gamma coactivator 1 alpha*	nDNA	FJ710605.1	Forward	caaccaccttgccacttcct
			Reverse	cggtgatcccttgtggtcat

#### 4.3.6. Quantitative Real-Time PCR

Reactions were performed in 96-Well Optical Reaction Plates (Applied Biosystems #4306737) using an ABI 7500 Real-Time PCR System (Applied Biosystems #4351104). The Sybr^®^ Green was used as the reporter dye and ROX as the passive reference according to the manufacturer’s protocol for Power Sybr^®^ Green Master Mix (Applied Biosystems #4367660). Each reaction consisted of 25 µL of master mix, 100 nM concentrations of both forward and reverse primers, 50 ng of cDNA template, and nuclease-free water to bring the final volume to 50 µL. Each gene/tissue combination contained three replicates per sample along with negative reverse transcriptase controls to check for contamination of RNA with genomic DNA and no-template controls to check for reagent contamination [48]. The amplification profile was 50 °C for 2 min, 95 °C for 10 min, and 40 cycles at 95 °C for 15 s and 60 °C for 60 s, followed by dissociation curve analysis to ensure purity of the amplicon. Relative transcript values were quantified from comparison of measured threshold cycle (*C*_t_) values for each target gene to the designated control housekeeping genes (*β*-*actin* and *ef1*α) and then to a calibrating sample using the 2^−ΔΔ*C*t^ method described by Livak and Schmittgen [49]. Relative quantification calculations were performed using Applied Biosystems 7500 software v2.0.2 with family 120 fed diet 40/10 (the treatment with high-FE fed the lowest dietary lipid level) serving as the calibrator for all the biological group comparisons (Family + Diet). Gene expression levels from all other treatments were expressed as fold change when compared with the calibrating treatment group.

### 4.4. Statistical Methods

Data were analyzed by two-way ANOVA to test the effects of the experimental diets, the families and the interaction diet x family. A significant level of *p* < 0.05 was used. When there were significant differences, the least significant difference procedure [50] was applied using Statistical Analysis System version 9.0 software (SAS Institute Inc., Cary, NC, USA). Each aquarium was used as an experimental unit.

## 5. Conclusions

This study for the first time evaluated the molecular basis of dietary lipid level on mitochondrial and nuclear genes involved in mitochondrial electron transport chain. This study demonstrated the association between the expression of mitochondrial and that of nuclear genes involved in electron transport chain in fish liver, intestine and muscle. This work also showed that the expression of mitochondrial genes is coupled with the expression of uncoupling proteins (UCP) in the liver and the intestine of rainbow trout. Therefore, the analysis of the molecular basis of mitochondrial function appears to be a promising pathway for the understanding of dietary lipid (and the other nutrients) utilization in fish, in order to formulate strong recommendations for the selection of high feed-efficient fish and for diet formulation in aquaculture. A further study conducted on the interaction between graded dietary protein and lipid levels on mitochondrial and nuclear gene expressions will be informative.
